# The Role of Fast-Cycling Atypical RHO GTPases in Cancer

**DOI:** 10.3390/cancers14081961

**Published:** 2022-04-13

**Authors:** Pontus Aspenström

**Affiliations:** Rudbeck Laboratory, Department of Immunology, Genetics and Pathology (IGP), Uppsala University, SE-751 85 Uppsala, Sweden; pontus.aspenstrom@igp.uu.se; Tel.: +46-18-4710000

**Keywords:** RHO GTPases, atypical RHO GTPases, fast-cycling RHO GTPases, oncogenes, actin dynamics, cell migration

## Abstract

**Simple Summary:**

For many years, cancer-associated mutations in RHO GTPases were not identified and observations suggesting roles for RHO GTPases in cancer were sparse. Instead, RHO GTPases were considered primarily to regulate cell morphology and cell migration, processes that rely on the dynamic behavior of the cytoskeleton. This notion is in contrast to the RAS proteins, which are famous oncogenes and found to be mutated at high incidence in human cancers. Recent advancements in the tools for large-scale genome analysis have resulted in a paradigm shift and RHO GTPases are today found altered in many cancer types. This review article deals with the recent views on the roles of RHO GTPases in cancer, with a focus on the so-called fast-cycling RHO GTPases.

**Abstract:**

The RHO GTPases comprise a subfamily within the RAS superfamily of small GTP-hydrolyzing enzymes and have primarily been ascribed roles in regulation of cytoskeletal dynamics in eukaryotic cells. An oncogenic role for the RHO GTPases has been disregarded, as no activating point mutations were found for genes encoding RHO GTPases. Instead, dysregulated expression of RHO GTPases and their regulators have been identified in cancer, often in the context of increased tumor cell migration and invasion. In the new landscape of cancer genomics, activating point mutations in members of the RHO GTPases have been identified, in particular in RAC1, RHOA, and CDC42, which has suggested that RHO GTPases can indeed serve as oncogenes in certain cancer types. This review describes the current knowledge of these cancer-associated mutant RHO GTPases, with a focus on how their altered kinetics can contribute to cancer progression.

## 1. Introduction

The RHO GTPases consist of a group of GTP-hydrolyzing enzymes that belong to the RAS superfamily of small GTPases. In human cells, there are 20 different members of the RHO GTPases that can be further divided into eight subgroups: RAC, RHO, CDC42, RND, RHOD/F, RHOU/V, RHOBTB, and RHOH (Table 1) [1,2]. Ever since the discovery of the first RHO gene, RHOB, in 1985, the RHO GTPases have been considered to have low oncogenic potential in vivo [3]. Mutant RHO variants have been shown to transform cells in various in vitro models, but these studies have relied mainly on laboratory-generated activating mutants of RHO GTPases, rather than on mutant proteins found in tumors. Instead, the prevailing view has been that the link between RHO GTPases and cancer is of a more indirect nature.

This view originated from a number of studies that demonstrated that certain members of the RHO GTPases, as well as their regulators and the components of their downstream signaling pathways, are differently expressed in a wide range of tumors, which suggests a role in cancer [4]. These views defined the scientific climate for a long time until, in 2012, cancer-associated activating mutants were identified in malignant melanoma [5,6]. In this article, I will describe the recent developments in the field of cancer-associated mutant RHO GTPases. I will describe the underlying mechanisms for their oncogenic properties, and in the process, I will describe the concept of atypical fast-cycling RHO GTPases. Finally, I will discuss the current views on how fast-cycling GTPases can contribute to cancer [7].

## 2. The Origin of the RHO GTPases

As already mentioned, the first RHO gene was discovered in 1985. This finding is a good example of the serendipitous nature of scientific progress. The scientist responsible for the discovery, Pascal Madaule, was at the time a post-doctoral fellow in Richard Axel’s research group at Columbia University in New York (USA). The project that Pascal was involved in aimed to clone an ortholog of the α subunit of human chorionic gonadotropin in the sea slug *Aplysia californica*. Unexpectedly, he instead identified a gene related to the human RAS genes, hence the name RAS homologous (RHO) [3]. The identification of the RHO genes was soon followed by the discoveries of a number of RHO-related genes, RHO, RAC, and CDC42 [8,9,10,11,12,13]. At this time, during the mid-1980s, the three RAS genes *H-RAS*, *K-RAS*, and *N-RAS* had been identified and demonstrated to be prominent oncogenes in several human cancers [14]. In contrast, similar oncogenic properties were not apparent for the RHO GTPases. Instead, several, by now classical, papers from the research group of Alan Hall during the early 1990s demonstrated that the RHO GTPases are key regulators in the signaling cascades that control actin dynamics in eukaryotic cells [15,16,17]. These studies paved the way for a paradigm, stating that only three RHO members, RHOA, RAC1, and CDC42, were sufficient to regulate the organization and dynamics of the actin filament system, and thereby complex cellular processes such as cell morphogenesis and cell migration [18]. Even if this model seems beautiful in its simplicity, it is a clear oversimplification; all 20 members of the RHO GTPases have both unique and overlapping roles in the regulation of a multitude of cellular processes [1,2].

According to the standard model, small GTPases alternate between tinactive, GDP-bound and active, GTP-bound conformations. This conformational change results in the exposure of structural elements at the surface of the active GTPase, which allows it to interact with other proteins, as so-called effectors, which serve as downstream recipients of a signaling cue [14]. Thus, small GTPases serve as binary molecular switches that are either in an OFF or an ON mode. Two categories of regulatory proteins, the guanine nucleotide exchange factors (or GEFs) and the GTPase activating proteins (or GAPs) tightly regulate the cycling between these two modes. The GEFs catalyze the nucleotide exchange in the active site from GDP to GTP, thereby serving as positive regulators. The GAPs catalyze the hydrolysis of GTP to GDP, thereby serving as negative regulators [14]. This standard model is also applicable to the RHO family of small GTPases. The human genome harbors around 70 RHOGEFs and 80 RHOGAPs [18,19]. In addition, there is yet another group of RHO regulators: the RHO GDP dissociation inhibitors (or RHOGDIs; there are three members of this protein family) [20]. This group of proteins sequesters RHO GTPases in the inactive GDP-bound conformation. Various activating stimuli result in separation of the two proteins, allowing the activation of RHO GTPases by the GEFs [20]. This control mechanism of RHO activity is an elegant construction by nature; however, it only applies to 10 of the RHO GTPases: the members of the classical subfamilies RHO, RAC, and CDC42. Importantly, the other 10 members do not follow this simple scheme of activation and are therefore referred to as atypical RHO GTPases [7].

Most RAS-like small GTPases contain a so-called CAAX box at their extreme C-terminus. This is a tetrapeptide motif with the consensus sequence: cysteine, followed by two aliphatic amino-acid residues, and a less defined amino-acid residue at the ultimate position [14]. The CAAX box undergoes posttranslational modifications through covalent attachment of an isoprenoid moiety at the cysteine residue, followed by cleavage of the AAX peptides and carboxymethylation of the resulting ultimate prenylated cysteine. The RAS GTPases are modified by a 15-carbon farnesyl, and of the farnesylated RAS proteins can thereby be targeted to lipid to the plasma membrane or to other intracellular lipid bilayers. In contrast, the RHO GTPases are most often modified by a 20-carbon geranylgeranyl moiety [21]. This modification is important for membrane targeting of RHO GTPases, but it is also required for their control by RHOGDIs. Again, this concept of activity control only applies to the classical 10 RHO GTPases; the atypical RHO GTPases do not undergo prenylation and do not bind RHOGDIs.

## 3. The Concept of Atypical RHO GTPases

The first indication of the existence of RHO GTPases with atypical properties came from the discovery of RHOE (also known as RND3) [22]. RHOE was shown to lack intrinsic GTPase activity and to reside exclusively in a GTP-bound conformation inside cells. In addition, RHOE was resistant to the influence of RHOGAPs. The GTPase deficiency turned out to be caused by differences in the amino-acid sequence at three key positions in the nucleotide-binding pocket: 12, 59, and 61 (following RAS numbering of the codons). At position 12, RHOE has a serine instead of glycine; at position 59, a serine instead of alanine; and at position 61, an aspartic acid instead of a glutamine. These amino-acid substitutions are known to be oncogenic in RAS because they render RAS GTPase-deficient [14]. Similar types of amino-acid substitutions can be found in all three RND proteins, as well as in RHOH and RHOBTB. These RHO subfamilies are therefore classified as GTPase-deficient RHO GTPases [23,24,25]. The GTPase-deficient RHO GTPases are not only resistant to RHOGAPs, they are also refractive to regulation by RHOGEFs and they do not bind RHOGDIs. This means that they are regulated by other mechanisms, for instance by posttranslational modifications, such as phosphorylation, or by regulation at the level of transcription [25].

A second group of atypical RHO GTPases comprises the fast-cycling atypical RHO GTPases, which include RHOD, RHOF, RHOU, and RHOV [6]. The GTPase activity is more or less intact in the fast-cycling RHO GTPases, but the intrinsic GDP/GTP exchange activity is greatly elevated, meaning that the exchange of the nucleotides at the active site occurs without the involvement of RHOGEFs [26,27,28]. Due to the roughly 10-fold higher intracellular levels of GTP over GDP in most cell types, the fast-cycling RHO GTPases will reside predominantly in an active conformation [29]. In contrast to the GTPase-deficient RHO GTPases, the amino-acid residues at positions 12, 59, and 61 in the fast-cycling RHO GTPases are the same as in the classical RHO GTPases. Similar to the GTPase-deficient RHO GTPases, no RHOGEFs, RHOGAPs, and RHOGDIs have been found for the fast-cycling RHO GTPases.

## 4. The Mechanisms Underlying an Increased Intrinsic Exchange Activity

**Figure 1:**
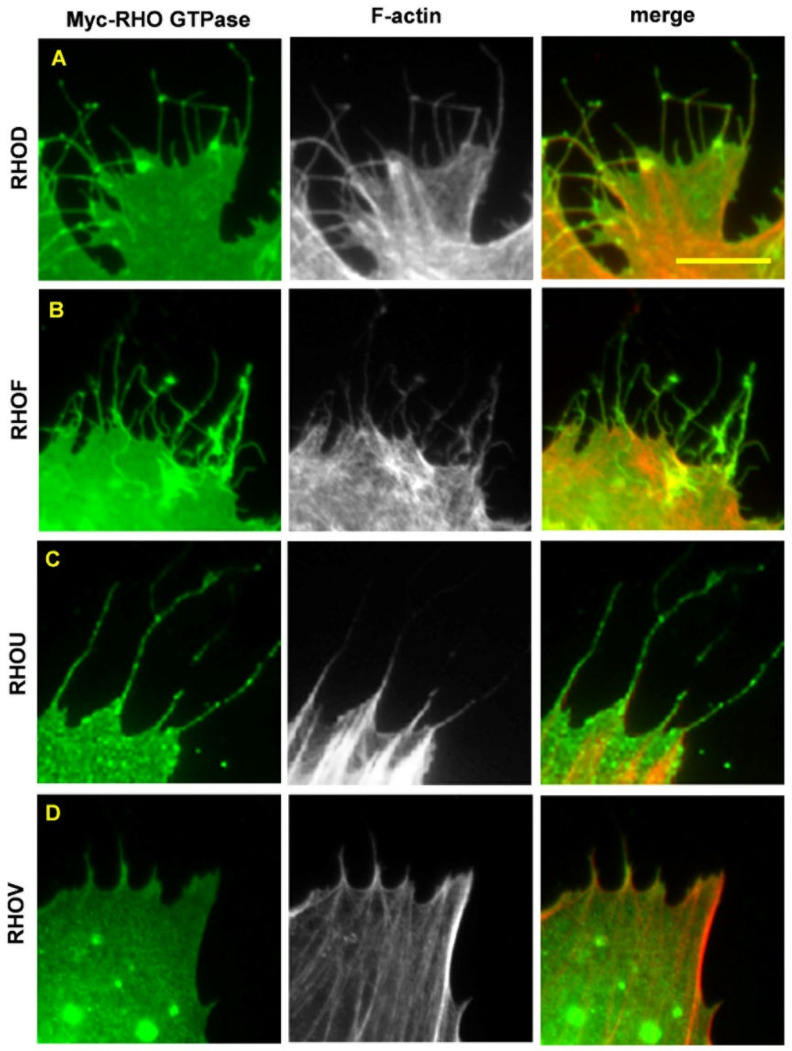
Atypical fast-cycling RHO GTPases trigger the formation of filopodia in fibroblasts. Human BJ/SV40T fibroblasts were transfected with Myc-RHOD (**A**), Myc-RHOF (**B**), Myc-RHOU (**C**), or Myc-RHOV (**D**). A non-transfected control is shown in Figure 3D for comparison. The Myc-tagged RHO GTPases were visualized using a mouse anti-Myc antibody, followed by an AlexaFluor 488-conjugated anti-mouse antibody. Filamentous actin was visualized with TRITC-conjugated phalloidin. Scale bar, 10 µm.

**Figure 2:**
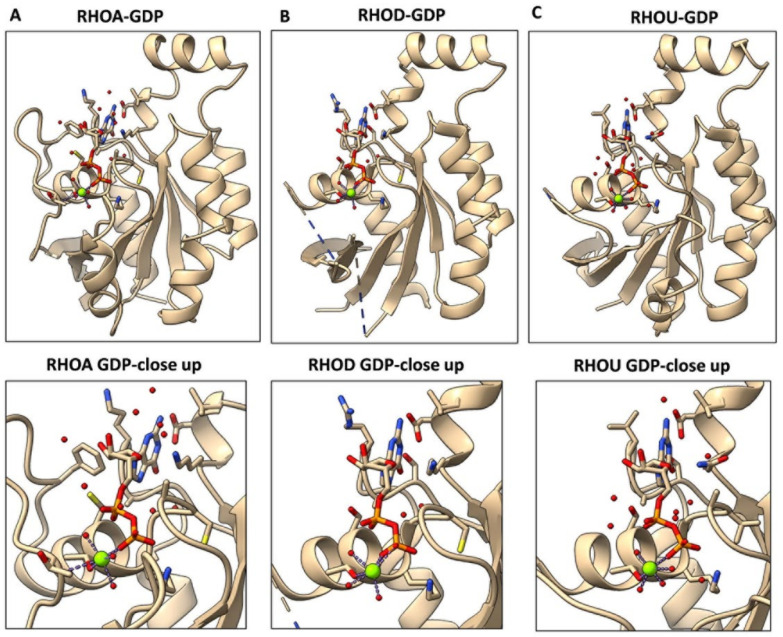
Three-dimensional structures of GDP-bound RHOA, RHOD, and RHOU. Structural information was collected from the PDB database, and are displayed for RHOA-GDP, 1FTN (**A**), RHOD-GDP, 2J1L (**B**), and RHOU-GDP, 2Q3H (**C**). Top: Overview. Bottom: Close-ups of GDP positioning. Images were generated with the USCF ChimeraX software (version 1.2, http://www.rbvi.ucsf.edu/chimerax) (version: 1.2.5 (24 May 2021).

RHOU (also known as WRCH1) was the first RHO member to be described with an elevated exchange activity, but with an intact GTPase activity [26,27]. Although RHOV has several characteristics that suggest that it is also fast cycling, the kinetics of RHOV have not yet been established. RHOU was originally identified as a gene responsive to Wnt-1; however, the possible role of RHOU in Wnt signaling is not clear [30,31]. RHOD and RHOF have been shown to be fast-cycling GTPases, although there are contradicting views on this for RHOF [28,32]. One common cellular response for all of these four RHO members is that when ectopically expressed in various cell models, they trigger the formation of filopodia (Figure 1) [4]. The mechanisms underlying the fast-cycling properties are not clear. RHOU and RHOV have tyrosines instead of phenylalanine residues in the positions equivalent to codon 28 in RAC1, which might lead to reduced hydrophobic interactions with the guanine base. RHOD and RHOF have phenylalanines in this position, similar to the classical RHO GTPases, but the amino-acid residues in proximity of this position differ from the classical RHO GTPases, which is likely to alter the nucleotide binding capacity of RHOD and RHOF (Figure 2) [28]. For further reading on signaling pathways and effectors downstream of RHOU and RHOV, please see the following recent review articles [7,31].

## 5. RHOU and RHOV in Cancer

No point mutations related to cancer have been reported for RHOU or RHOV to date. Instead, increased expression of these RHO members has been linked to cancer progression. This is not so surprising, as the fast-cycling RHO GTPases have been shown to be constitutively active in their wild-type forms, so increased expression is likely to result in more active protein [6]. Elevated expression of RHOV has been shown in lung adenocarcinoma and correlated with high frequency of metastasis. Moreover, ectopic expression of RHOV in the A549 lung cancer cell line resulted in increased wound closure and focus formation [33]. Knock-down of RHOV in this same cell line resulted in reciprocal effects: slightly decreased wound closure and focus formation. Another study, which also analyzed expression levels of all 20 of the RHO members, concluded that RHOV was specifically overexpressed in lung adenocarcinoma [34]. A link to lung cancer was furthermore suggested in a study on non-small-cell lung cancer tumors in which the RHOV transcript was reported to be overexpressed in cell lines and in patient material [35]. Together, these studies have suggested a role for RHOV in lung cancer, but how RHOV functionally contributes to cancer progression is not known at present.

RHOU has a more ubiquitous expression pattern compared to RHOV [36]. Several studies have shown that RHOU is up-regulated in various cancers, such as T-cell acute lymphoblastic leukemia, prostate cancer, multiple myeloma, and breast cancer [37,38,39,40,41,42]. Knock-down of RHOU in T lymphoblastoid cells resulted in decreased cell migration and chemotaxis towards CXCL12 [37]. Increased expression of fatty-acid synthase in prostate cancer is associated with tumor progression. Fatty-acid synthase expression positively regulates cell migration in a RHOU-activation-dependent manner by regulation of the levels of RHOU palmitoylation [38]. In multiple myeloma cells, decreased RHOU expression using siRNA reduced their cell migration [41]. Additionally, in breast cancer cells, depletion of RHOU expression resulted in impaired cell migration and invasion. As atypical RHO GTPases are believed to be regulated at the level of transcription, it is relevant to postulate that increased expression of RHOU and RHOV is associated with increased activity of these proteins. However, RHOU expression has not only been positively correlated with tumor progression and increased cell migration. A recent study showed that RHOU expression is decreased in human colorectal tumor samples. Furthermore, studies in mice showed that cells in the gut of RHOU knock-out mice had an increased migratory capacity [43]. RHOU expression can therefore give rise to different cellular responses depending on the cellular context. Clearly more studies are needed to unravel the signaling capacity of RHOU.

## 6. RHOD and RHOF in Cancer

RHOD and RHOF have both been shown to function as fast-cycling RHO GTPases [28]. Another study came to a conflicting conclusion, however, as it suggested that the intrinsic GDP/GTP exchange activity of RHOF is slow, rather than fast [32]. The reason for this dichotomy is difficult to evaluate but might be because different methods were used for protein purification. Moreover, in the latter study, RHOF was not compared side by side to other RHO proteins [32].

Again, no cancer-related point mutations have been reported for RHOD or RHOF, although they have both been shown to be differently expressed (mostly overexpressed) in cancer. In acute myeloid leukemia, high expression of RHOF is associated with reduced overall survival [44]. Furthermore, RHOF was shown to be frequently up-regulated in hepatocellular carcinoma, and increased RHOF expression is associated with poor clinical outcome. Finally, RHOF up-regulation markedly increased in vivo cell migration and invasion of human hepatocyte carcinoma HepG2 cells [45].

## 7. Classical RHO GTPases as Proto-Oncogenes

The oncogenic properties of RHO GTPases have been debated since their respective discoveries. The general view has stipulated that RHO GTPases per se are not oncogenic, but that the RHOGEFs might serve as bona fide oncogenes [46,47]. Several studies have implicated that the constitutively active mutants of RHOA and RAC1 RHOA/G14V and RAC1/G12V, have transforming properties in several of the classical in vitro assays for cell transformation (e.g., focus formation assays, growth in soft agar) [48,49,50]. In addition, several observations have indicated that constitutively active mutants of RHOA and RAC1 can cause tumor growth in nude mice [48]. However, these results were dependent on laboratory-generated mutant RHO GTPases, and no cancer-associated mutated RHO GTPases were reported for a long time.

**Figure 3:**
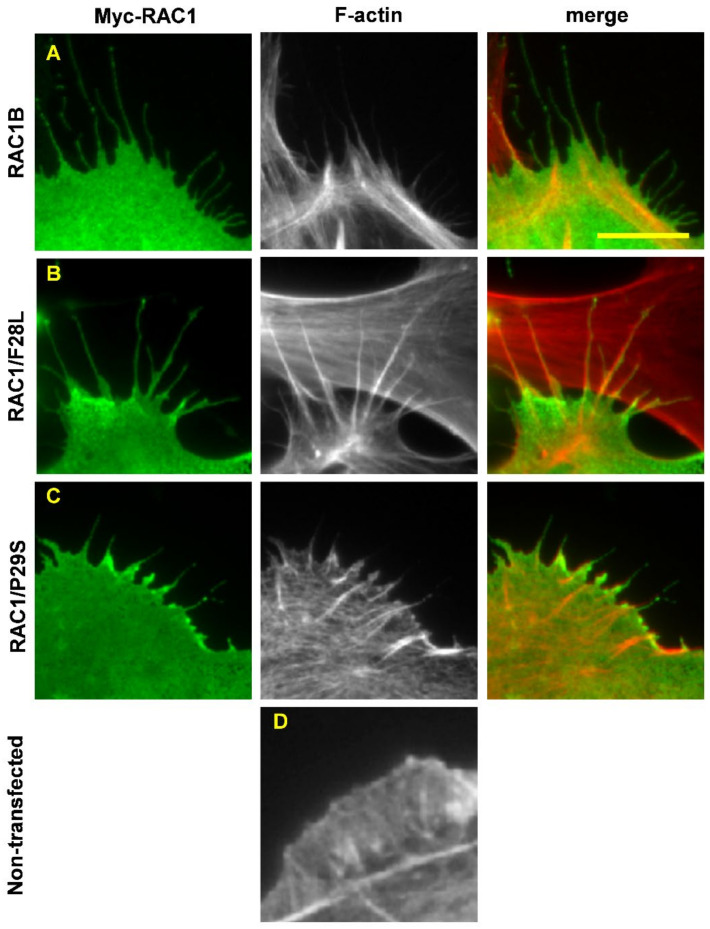
Fast-cycling RAC1 mutants trigger the formation of filopodia in fibroblasts. Human BJ/SV40T fibroblasts were transfected with Myc-RAC1B (**A**), Myc-RAC1/F28L (**B**), Myc-RAC1/P29S (**C**), or a non-transfected control (**D**). Myc-tagged RAC1 variants were visualized using a mouse anti-Myc antibody, followed by an Alexa Fluor 488-conjugated anti-mouse antibody. Filamentous actin was visualized with TRITC-conjugated phalloidin. Scale bar, 10 µm.

**Figure 4:**
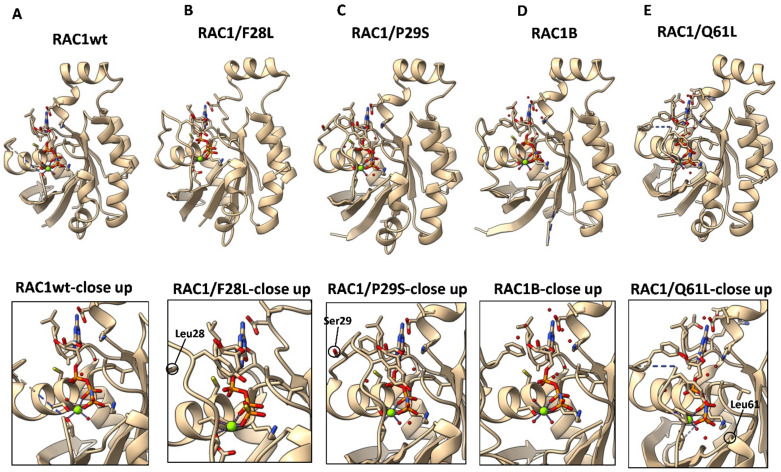
Three-dimensional structures of GDP-bound RAC1 variants. Structural information was collected from the PDB database. RAC1wt-GDP, 3TH5 (**A**); RAC1/F28L-GDP, 4GZM (**B**); RAC1/P29S-GDP, 3SBD (**C**); RAC1B-GDP, 1RYH (**D**); and RAC1/Q61L-GDP, 4GZL (**E**). Top: Overview. Bottom: Close-ups of GDP positioning. Images of the three-dimensional structures were generated with the USCF ChimeraX software (version 1.2, http://www.rbvi.ucsf.edu/chimerax) (version: 1.2.5 (24 May 2021).

However, modern DNA-sequencing tools that allow large-scale sequencing of entire cancer genomes has now dramatically changed the scene, and the first step to an altered view on RHO GTPases as oncogenes came from the identification of a recurrent somatic point mutations in RAC1 (RAC1/P29S) in a sun-exposed melanoma, in 2012 [5]. Already before this finding, a splice variant of RAC1, RAC1B, was reported to have transforming capacity. Importantly, these mutant RAC1 variants were shown to function as fast-cycling RHO GTPases [51,52,53] (Table 2). Another similarity to the atypical fast-cycling RHO members is that these fast-cycling mutants of RAC1 promote the formation of filopodia (Figure 3) [54]. Comparisons between the three-dimensional structures of wild-type RAC1 and RAC1B and RAC1/P29S reveals that there are clear differences in the orientations of the key amino-acid residues in their interactions with the guanine nucleotides (Figure 4).

### 7.1. RAC1/P29S

A point mutation in codon 29 of RAC1, or rather in the *Caenorhabditis* *elegans* ortholog CED-10, was first identified in a screening for mutant genes that confer synthetic lethality with a weak ced-10 mutant. This screening identified a P29L mutant of CED-10 [68]. Later, the occurrence of a point mutation in human RAC1 was revealed in a study of the mutational landscape in melanoma tumors. This study involved 147 tumor samples and identified mutations in *BRAF* and *NRAS* at high incidence, as expected. Interestingly, in sun-exposed melanomas, the third most common recurring somatic mutation was a serine for a proline mutation in *RAC1* [5]. This RAC1/P29S mutated protein was shown to cause increased GTP-binding associated with increased interactions with two previously known RAC1 effectors, PAK1 and MLK3. Furthermore, forced expression of RAC1/P29S in melanocytes resulted in increased cell proliferation and motility in a Boyden-type migration assay. Transient transfection of EGFP-tagged RAC1/P29S in COS7 cells resulted in increased accumulation of the mutant RAC1 protein in dorsal membrane ruffle-like structures [5]. The kinetics of RAC1/P29S were further analyzed in a study by Davies et al. [53], which demonstrated that RAC1/P29S is a fast-cycling RHO GTPase. These kinetic properties were similar to RAC1/F28L, a laboratory-generated mutant RHO GTPase that is also fast-cycling [55]. However, detailed structural analysis indicated that the fast-cycling of RAC1/F28L was the result of reduced interactions between the guanosine ring and mutated phenylalanine 28, whereas the fast-cycling of RAC1/P29S appears to result from destabilization of the GDP-bound conformation (Figure 4) [53]. Here, Davies et al. [53] also reported that RAC1/P29S increased membrane ruffling in transiently expressed COS7 cells as well as in NIH3T3 fibroblasts stably expressing RAC1/P29S. However, a study that compared the three-dimensional structures of RAC1/P29S and RAC1/A159V came to a somewhat different conclusion [69]. The authors to this report suggested that the fast-cycling of RAC1/P29S is the result of an open conformation of the switch I motif, and that the interaction between GTP and phenylalanine 28, proline 29 (which is now a serine in the mutant protein) and glycine 30 was lost [69].

The effects of fast-cycling RAC1 mutants on membrane ruffling are in contrast to a study of the cellular effects triggered by a panel of CDC42 and RAC1 mutants [54]. In this study, it was shown that fast-cycling mutants of CDC42 and RAC1 (including RAC1/F28L and RAC1/P29S) trigger the formation of filopodia in human fibroblasts as well as in porcine aortic endothelial cells. In contrast, GTPase-deficient mutants of CDC42 and RAC1 induce the formation of lamellipodia (Figure 3) [54]. The reason for this discrepancy was not clear, but COS7 cells do not represent an ideal model system for the analysis of cytoskeletal reorganization, and the generation of NIH3T3 cells stably expressing RAC1/P29S mutants might trigger compensatory mechanisms that can confound the acute effects on actin organization. Such a cell-type-dependent response was indeed supported by a study by Mohan et al. [70], where they showed that expression of RAC1/P29S in melanoma A375 cells induced the formation of extended lamellipodia driven by dendritic actin networks. Interestingly, the RAC1/P29S-induced cell proliferation was dependent on the integrity of the actin network. Moreover, the formation of extended lamellipodia resulted in sequestration and inactivation of the tumor suppressor Merlin/NF2 in a mechanism that involved phosphorylation of serine 518 on Merlin/NF2 by PAK1 [70]. This finding is in agreement with the RAC1/P29S-dependent increase in PAK1 activity described previously [5].

The studies described thus far have mainly described the cellular functions of RAC1/P29S in vitro, so what about the in vivo functions? RAC1/P29S was shown to regulate the expression of ‘Programmed death-ligand 1’ (PD-L1), which is an immune regulatory molecule [71]. PD-L1 up-regulation might allow cancers to evade immune control, and it is often associated with increased tumor aggressiveness [72]. Recurrent RAC1/P29S in primary cutaneous melanomas has often been shown in conjunction with mutant *BRAF*, and possibly aggravates BRAF-dependent disease progression [73]. Studies in mice demonstrated that RAC1/P29S can serve as an oncogenic driver mutation. Ubiquitous expression of RAC1/P29S at endogenous levels in adult mice resulted in B-cell lymphoma [74]. RAC1/P29S expression alone in mouse melanocytes did not result in melanoma; however, in combination with mutant BRAF, RAC1/P29S expression resulted in melanoma in these mice. The most plausible mechanism for RAC1/P29S in tumor progression is through activation of the serum response factor/myocardin-related transcription factor (SRF/MTRF) transcriptional programs, which lead to mesenchymal transition of melanocytes [74].

Some additional cancer-associated mutants in RAC1 and RAC2 have been reported in common cell lines and are given in public databases [61]. This way, two new fast-cycling mutants of RAC1 were identified, RAC1/N92I and RAC1/C157Y; furthermore, they were shown to undergo in vitro transformation [61]. However, not much else is known about these mutant proteins and how they contribute to human cancer.

### 7.2. RAC1B

The first observation that suggested that oncogenic RHO GTPases do exist outside the test-tube came from the discovery of a splice variant of RAC1, called RAC1B. This is not a recurrent somatic mutation, but rather a splice variant present at low levels in many human tissues, and it was noted for colorectal cancer [51]. RAC1B results from an alternative splicing event that adds 57 extra nucleotides between codons 75 and 76, which results in 19 extra amino-acid residues immediately behind the switch II motif [51,52]. Importantly, studies of its kinetics revealed that RAC1B has fast-cycling properties [75]. RAC1B was shown not to bind RHOGDI, and in contrast to RAC1/P29S, not to interact with PAK1, or at least, the interaction with full-length PAK1 appears to be abolished [76,77]. The binding spectra of all of the RAC1 mutants are not completely known, but it is clear that RAC1B has a different affinity for many of the RAC1 effectors identified, and triggers other downstream pathways compared to wild-type RAC1 [6]. For instance, RAC1B does not activate NF-κB or cyclin D1 expression but can trigger the AKT signaling pathway [77].

Several studies have indicated that increased RAC1B expression is associated with cellular transformation in commonly used cell models [77,78]. RAC1B is not involved in the formation of lamellipodia but is involved in the formation of filopodia [54,78]. What is the mechanism underlying the effects on cytoskeletal organization and cell morphology? One model suggests that RAC1B interferes with RAC1 signaling, as forced expression of a GTPase-deficient mutant of RAC1B (RAC1B/G12V) results in loss of endogenous RAC1 at peripheral membranes and an increase in activated RHOA [78]. According to this model, the RAC1B-induced cellular transformation will be dependent on RHOA activity. However, RAC1B does not appear to trigger all of the RHOA-dependent cellular effects, e.g., it does not appear to trigger the formation of stress fibers. An interesting study showed that MMP-3-induced cell transformation requires RAC1B [79], where they also showed that MMP-3 activation resulted in epithelial–mesenchymal transition (EMT) in mouse breast epithelial cells. MMP-3 induction was associated with activation of RAC1B, as well as with its increased expression, which resulted in ROS formation followed by Snail1 expression and EMT [79].

What is the function of RAC1B, and is it only expressed during disease? Studies on the evolutionary aspects of RAC1B have demonstrated that the exon involved in the alternative splicing can be found exclusively in amniotes, so it is possible that RAC1B has a role in a normal cellular context [36]. Additionally, while RAC1B has cell-transforming properties at the cellular level, what is its role in cancer? This is still an open question, and one role suggested by Nimnual et al. would be its modulation of the function of the major splice variant, or to shift the balance between RAC1- and RHOA-dependent signaling [78]. There are several reports on RAC1B expression in human cancers, such as breast, colorectal, lung, thyroid, and pancreatic cancers [51,52,56,57,58,59,60]. However, many of these studies are based on cellular models and associations, rather than on hard data that univocally puts RAC1B as a causative factor in tumor progression. An important step to define a mechanism for RAC1B in progression of colorectal cancer comes from a recent study by Gudiño et al. [80]. They showed that high *RAC1B* expression correlates with high WNT activity and poor prognosis. In a mouse model for colorectal cancer, it was shown that abrogating *Rac1b* resulted in significantly decreased tumor burden and increased overall survival of the mice. This study also demonstrated the presence of a hitherto undiscovered role of RAC1B in EGF receptor trafficking. Deletion of *Rac1b* resulted in decreased EGFR internalization and increased receptor degradation through lysosomal sorting. Thus, increased *Rac1b* expression was associated with increased EGFR signaling. Interestingly, in studies using patient-derived organoids, it was shown that tumors resistant to EGFR inhibitors (e.g., cetuximab) were sensitive to RAC1B depletion, which suggests that this strategy might be used in a clinical setting [80].

The studies discussed thus far indicate the tumor-promoting role of RAC1B. However, there are indications that RAC1B might serve as a tumor suppressor, in particular in the context of TGFβ signaling [81,82]. Studies in pancreatic cancer cells showed increased TGFβ-dependent cellular responses, such as activation of the MKK6/p38 and MEK/ERK signaling pathways in cells where RAC1B was ablated. In addition, TGFβ-induced expression of EMT marker genes and the morphological cell alterations associated with EMT were much more pronounced in cells lacking RAC1B [83]. Thus, there is support for RAC1B as a tumor-promoting as well as a tumor-suppressing factor, and the reason for this apparent dichotomy is not clear at the moment. RAC1B has been shown to modulate RHOA signaling; however, the effect on the activity of the additional 18 RHO GTPases expressed in human tissues is not known. More studies are clearly needed to clarify the picture and reveal the molecular functions of RAC1B in more detail.

## 8. RHO Mutants in Cancer

Similar research strategies that resulted in the identification of the fast-cycling mutants of RAC1 have resulted in the identification of somatic mutations in RHOA (Table 2). For instance, mutations in RHOA were identified in angioimmunoblastic T-cell lymphoma, peripheral T-cell lymphoma, adult T-cell leukemia/lymphoma, and diffuse-type gastric carcinoma [62,63,64,65,66]. The most common RHOA mutation in these datasets was substitution of a valine for a glycine at codon 17 (RHOA/G17V), which appeared at high incidence, although other mutations were also identified [6,62,63,84].

RHOA/G17V was not defined as a fast-cycling RHO GTPase; instead, it has similar kinetics as the classical dominant-negative RHOA variant RHOA/T19N. This mutant protein has decreased affinity for guanosine nucleotides and higher affinity for RHOGEFs, and it is thought that dominant-negative RHO GTPases sequester RHOGEFs, and thereby block the downstream signaling. Expression of RHOA/G17V in T Jurkat cells resulted in increased cell proliferation and invasion, properties that are indicative of cell transformation. The underlying mechanism is not entirely clear here, but presumably this down-regulation of RHO-dependent signaling results in increased RAC1 signaling. Interestingly, expression of RHOA/G17V in NIH3T3 fibroblasts or HeLa cells resulted in loss of stress fibers and formation of filopodia-like protrusions, a response that resembles the phenotype induced by the expression of fast-cycling RHO GTPases [63,65]. In addition to this panel of dominant-negative variants of RHOA, two mutations, RHOA/C16R and RHOA/A161P, have been described as fast-cycling variants of RHOA [64], with little known of the roles of these two mutant proteins in a clinical setting.

## 9. Additional Cancer-Associated Mutations in RHO GTPases

There are sporadic observations of cancer-associated point mutations in other RHO GTPases (Table 2). Somatic mutations in CDC42 have been identified in well-differentiated papillary mesothelioma, where two different CDC42 mutants were detected: CDC42/P34Q and CDC42/Q61R [67]. The nucleotide-binding characteristics of these mutants have, to date, not been analyzed, but the CDC42/Q61L mutant is known to have defective GTPase activity. The proline at codon 34 resides in the so-called effector-binding loop, and it is therefore expected to result in altered binding to effector proteins, and thereby to altered downstream signaling [85].

## 10. Summary

Although oncogenic mutations in RHO GTPases are rare, there clearly remain many more to be characterized (e.g., see Catalogue of Somatic Mutations in Cancer database; https://cancer.sanger.ac.uk/cosmic). In addition, although oncogenic mutations in RHO GTPases are not common in a global setting, they might still have major impact on certain tumor types and might thus serve as ‘druggable’ targets in this context. A promising example is the combinatory treatment of malignant melanoma with B-RAF inhibitors and SRF/MTRF inhibitors in a mouse model of malignant melanoma [73]. Another example was the finding that tumors resistant to EGFR inhibitors are sensitive to RAC1B depletion. Hopefully, we will see more examples of this type in the future, and that this type of treatment regimen will eventually reach the clinic setting.

## 11. Conclusions

Research during the last couple of years has resulted in a paradigm shift and has bestowed RHO GTPases, in particular RHO GTPases with fast-cycling properties, with key roles in human cancer. A substantial number of cancer-associated point mutations in predominantly RAC1, RHOA, and CDC42 have been found and characterized. The kinetic properties of the oncogenic mutations in RHO GTPases differ from mutations in RAS; the former are most often fast-cycling and the latter GTPase deficient. These properties could make the oncogenic RHO GTPases, and/or signaling pathways regulated by this category of RHO GTPases, potential targets for future cancer treatments.

## Figures and Tables

**Table 1:** Subgroup divisions for the 20 different members of the RHO GTPases in human cells.

RHO Subfamily	Member	Catalytic Type	Kinetics and Enzymatic Activities
RAC	RAC1	Classical	Classical RHO GTPases have intact enzymatic activities and can cycle between GTP-bound and GDP-bound conformations
	RAC2		
	RAC3		
	RHOG		
RHO	RHOA		
	RHOB		
	RHOC		
CDC42	CDC42		
	RHOJ (TCL)		
	RHOQ (TC10)		
RND	RND1	GTPase deficient	GTPase deficient RHO GTPases cannot hydrolyze GTP
	RND2		
	RND3/RHOE		
RHOH	RHOH		
RHOBTB	RHOBTB1		
	RHOBTB2/DBC		
RHOD/F	RHOD	Fast-cycling	Fast-cycling RHO GTPases can freely cycle between GTP-bound and GDP-bound conformations without the involvement of RHOGEFs
	RHOF		
RHOU/V	RHOU(WRCH1)		
	RHOV(CHP)	Fast-cycling (?)	RHOV has not been confirmed to function as a fast-cycling RHO GTPase; however, its similarity to RHOU suggests that it is.

**Table 2:** Mutant RHO GTPases mentioned in the text.

Mutant Protein	Catalytic Type	Cancer Type	Ref
RAC/P29S	Fast-cycling	Sun-exposed melanoma	[5]
RAC1/F28L	Fast-cycling	Laboratory-generated mutant	[55]
RAC1B	Fast-cycling	Colorectal cancer	[51,56]
		Breast cancer	[52]
		Lung cancer	[57,58]
		Pancreatic cancer	[59]
		Thyroid cancer	[60]
RAC1/N92I	Fast-cycling	Fibrosarcoma cell-line HT1080	[61]
RAC1/C157Y	Fast-cycling	Lung cancer	[61]
RHOA/G17V	Decreased affinity for guanosine nucleotides	Angioimmunoblastic T-cell-lymphoma	[62,63]
		Adult T-cell leukemia/lymphoma (ATLL)	[64]
		Peripheral T-cell lymphoma	[65]
		Diffuse-type gastric carcinoma	[66]
RHOA/C16R	Fast-cycling	ATLL	[64]
RHOA/A161P	Fast-cycling	ATLL	[64]
CDC42/P34Q	Effector-binding?	Well-differentiated papillary mesothelioma	[67]
CDC42/Q61R	GTPase-deficient?	Well-differentiated papillary mesothelioma	[67]
